# Acoustic Correlates of Auditory Object and Event Perception: Speakers, Musical Timbres, and Environmental Sounds

**DOI:** 10.3389/fpsyg.2019.01594

**Published:** 2019-07-17

**Authors:** Mattson Ogg, L. Robert Slevc

**Affiliations:** ^1^Neuroscience and Cognitive Science Program, University of Maryland, College Park, College Park, MD, United States; ^2^Department of Psychology, University of Maryland, College Park, College Park, MD, United States

**Keywords:** auditory object, acoustics, timbre, speaker identification, environmental sound

## Abstract

Human listeners must identify and orient themselves to auditory objects and events in their environment. What acoustic features support a listener’s ability to differentiate the great variety of natural sounds they might encounter? Studies of auditory object perception typically examine identification (and confusion) responses or dissimilarity ratings between pairs of objects and events. However, the majority of this prior work has been conducted within single categories of sound. This separation has precluded a broader understanding of the general acoustic attributes that govern auditory object and event perception within and across different behaviorally relevant sound classes. The present experiments take a broader approach by examining multiple categories of sound relative to one another. This approach bridges critical gaps in the literature and allows us to identify (and assess the relative importance of) features that are useful for distinguishing sounds within, between and across behaviorally relevant sound categories. To do this, we conducted behavioral sound identification (Experiment 1) and dissimilarity rating (Experiment 2) studies using a broad set of stimuli that leveraged the acoustic variability within and between different sound categories via a diverse set of 36 sound tokens (12 utterances from different speakers, 12 instrument timbres, and 12 everyday objects from a typical human environment). Multidimensional scaling solutions as well as analyses of item-pair-level responses as a function of different acoustic qualities were used to understand what acoustic features informed participants’ responses. In addition to the spectral and temporal envelope qualities noted in previous work, listeners’ dissimilarity ratings were associated with spectrotemporal variability and aperiodicity. Subsets of these features (along with fundamental frequency variability) were also useful for making specific within or between sound category judgments. Dissimilarity ratings largely paralleled sound identification performance, however the results of these tasks did not completely mirror one another. In addition, musical training was related to improved sound identification performance.

## Introduction

Successful perception of speech (Creel and Bregman, 2011), music (McAdams and Giordano, 2009) and auditory scenes (Bregman, 1990; Bizley and Cohen, 2013) relies on identifying sound sources. That is, a listener’s perceptual and cognitive systems must transform acoustic signals into mental constructs, which help organize information from the environment. Regardless of the specific kind of sound one hears (whether it is speech, music, or another environmental sound) this process appears to involve (1) a multidimensional set of acoustic cues, (2) a process of matching cues to representations of objects and events stored in memory, and (3) the use of those object representations to organize auditory scenes (McAdams, 1993). While a large and important body of work has examined the perception and psychophysics of different acoustic attributes (Moore, 2012) it is still unclear how specific acoustic qualities contribute to the formation of auditory object and event representations for the wide array of natural stimuli relevant to human listeners. A better understanding of this process can help improve our knowledge of how humans navigate and interact with their auditory environment. This in turn might improve machine intelligence algorithms and assistive hearing therapies, both of which are increasingly vital for our aging and technology-immersed society.

Our current understanding of auditory object and event perception has been greatly advanced by the analysis of metrics that code differences among pairs of items. Dissimilarity data can come directly from behavioral ratings provided by participants (the more dissimilar two stimuli are, the higher the dissimilarity rating; Giordano et al., 2011), or from confusion rates in identification tasks (the more similar two stimuli are, the higher the degree of confusion; Giordano and McAdams, 2010; Sell et al., 2015). Such approaches provide rich data that often come in the form of a matrix representing how dissimilar all the possible pairs of items are within a set (or of interest for a particular study). Links can then be made between a listener’s perception (ratings or confusions) and the physical qualities of the stimuli they observed. This can be uncovered by correlating the physical attributes of the items with their positions in a derived multidimensional space (Shepard, 1980), or by directly correlating the observed matrix of dissimilarities with other item-pair-level dissimilarity matrices that correspond to differences among the stimuli in terms of their physical qualities or computational representations (Kriegeskorte and Kievit, 2013). The former analysis is known as multidimensional scaling (MDS) and the latter is known as representational similarity analysis (RSA). Thus, these approaches help provide an understanding of what stimulus characteristics participants use to perceive, represent, and distinguish complex natural stimuli.

Multidimensional scaling and RSA have typically been employed in the study of specific subsets of sound stimuli in isolation from one another (e.g., studies of speech sounds *or* of musical instrument timbres). Because of this, inferring what features are useful for natural sound perception *across* categories requires comparing findings across studies that involve different methods and participants. This presents two fundamental roadblocks to furthering our understanding of human auditory object and event perception, which our work addresses. First, it is currently difficult to determine what features are most influential for auditory object and event perception across (e.g., features useful for comparing instrument vs. instrument sounds *and* comparing speech vs. speech sounds) or between different sound categories (e.g., features useful for comparing instrument vs. speech sounds), and it is unclear what the relative level of importance of different acoustic features is in each of these contexts. Second, the predominant focus on *individual* sound classes in the literature obscures potentially important perceptual cues for distinguishing *between* categories of sound. As we demonstrate here, these between category distinctions are important for organizing how the listener distinguishes sounds and this process relies on specific cues that are not obvious from examining individual sound categories. An improved understanding of how stimuli from different sound categories relate to one another (1) could help inform interpretations of neural data or category specific cognitive processes, (2) could support the generation of new hypotheses regarding general perceptual mechanisms of natural sound perception and (3) could help illuminate valuable acoustic processing stages that cut across different domains of auditory research (see Ogg and Slevc, 2019, for a review).

### The Acoustic Basis of Auditory Object and Event Perception Within Different Categories of Sound

Dissimilarity-rating and identification (or confusion) data have been particularly useful in furthering our understanding of the perception of different musical instrument sound sources (Giordano and McAdams, 2010; Siedenburg et al., 2016b), or the musical quality known as timbre (McAdams and Giordano, 2009; Siedenburg and McAdams, 2017). Distinguishing among instruments has been shown to depend on two primary acoustic dimensions: a temporal dimension related to a sound’s attack or onset time (often its logarithm) and a spectral dimension related to the center of gravity, or centroid, of a sound’s frequency spectrum (Iverson and Krumhansl, 1993; McAdams et al., 1995; Lakatos, 2000). The degree of variability (or “flux”) in a sound’s frequency spectrum (Grey, 1977; Grey and Gordon, 1978) as well as the attenuation of even numbered harmonics have also been found to influence timbre perception (McAdams et al., 1995), albeit less consistently (Caclin et al., 2005).

Investigations of vocal sounds (including speech and speaker perception) have also relied on dissimilarity data. Speech is a rich auditory signal that involves a high level of acoustic redundancy. This allows a large degree of lexical (linguistic), phonetic and indexical (speaker-identifiable) information to be conveyed in a limited amount of time, such as within the few hundreds of milliseconds of a vowel (Fox et al., 1995; Iverson and Kuhl, 1995; Owren and Cardillo, 2006). Vowels are differentiated by the spectral positions of their formants, which are intentionally controlled during speech production by vocal tract changes. These vocal tract changes alter the spectral shape of the signal generated by a speaker’s vocal folds, or glottis (Hillenbrand et al., 1995; Ladefoged and Johnson, 2015). The make up of a speaker’s vocal tract and vocal folds are also constrained by that individual’s unique physiology, which imparts cues in the speech signal that are useful for speaker identification (Bachorowski and Owren, 1999; Johnson, 2005; Smith and Patterson, 2005). However, the same acoustic redundancy that makes speech so robust to acoustic distortion or interference (Shannon et al., 1995) poses some difficulty for defining the set of specific cues that support a listener’s speaker identification abilities, since the parameter space is very high. That is, not every cue that could potentially identify a given speaker needs to be used at the same time, nor are the same cues even used consistently by the same listener (Kreiman et al., 1992). Nonetheless, findings based on dissimilarity data and acoustic analyses suggest that speaker identification relates to fundamental frequency (determined by the vocal folds), formants in the frequency spectrum (determined by the vocal tract), and spectral slope (Matsumoto et al., 1973; Murry and Singh, 1980; Van Dommelen, 1990; Baumann and Belin, 2010; Sell et al., 2015). These are often simplified to glottal (source) and vocal tract (resonant) dimensions (Matsumoto et al., 1973; Baumann and Belin, 2010). Meanwhile, the vowels within each speaker are distinguished from one another based the different spectral positions of formants (Hillenbrand et al., 1995; Johnson, 2005; Ladefoged and Johnson, 2015).

Successful navigation of the environment also requires quickly recognizing (and potentially reacting to) a great variety of objects and events beyond music and speech. Indeed, investigations of such sounds from a typical human environment (which we will refer to as human-environmental sounds) frequently involve diverse sets of sound stimuli encompassing anything from mechanical or tool sounds to animal vocalizations and background noises (Ballas, 1993; Giordano et al., 2010; Kaya and Elhilali, 2014; Huang and Elhilali, 2017). Gygi et al. (2004, 2007) used identification and dissimilarity-rating data to find that different environmental sounds can be distinguished based how the sounds vary in their periodicity, loudness, spectral and temporal envelopes and the overall degree of change in the frequency spectrum. Hjortkjær and McAdams (2016) also found that the perception of the material that generated an environmental sound was related to the sound’s spectral envelope (spectral centroid), while the perception of the sound’s action was related to its temporal envelope (temporal centroid). Indeed, perception of both the action and resonating material of a sound appear to be the primary drivers of environmental sound perception (Gaver, 1993; Lemaitre and Heller, 2012, 2013).

Finally, a related body of work has illustrated the importance of joint spectral and temporal variability rates, called modulation power spectra, in timbre (Elliott et al., 2013; Thoret et al., 2016, 2017), speech (Elliott and Theunissen, 2009; Venezia et al., 2016) and natural sound perception (Singh and Theunissen, 2003; Theunissen and Elie, 2014). These powerful representations summarize rates of change in the sound’s spectrogram and appear to be similar to computations carried out in early acoustic processing stages in auditory cortex (Chi et al., 2005; Patil et al., 2012; Theunissen and Elie, 2014).

As noted above, these findings are predominantly based on separate studies that focused on individual categories. Only a few studies have examined multiple categories of sound together; however, these were either limited in their scope so as to focus only on two sound categories (speech and music Chartrand and Belin, 2006; Agus et al., 2012; Suied et al., 2014), or were not designed to compare sounds among these categories to facilitate an analysis of what cues were influential within and between different classes (Gygi et al., 2004, 2007; Bigand et al., 2011; Ogg et al., 2017). Again, this obscures an understanding of how different acoustic qualities might be important in differentiating natural sounds across or particularly between different categories.

### The Current Investigation: Auditory Object and Event Perception Across Sound Categories

The present studies aimed to relate previous work on individual domains of auditory research to one another via dissimilarity ratings of sounds from multiple categories. Like previous work, participants in this study were presented with a pair of sounds on each trial and were asked to simply rate how dissimilar the two sounds in the pair were from one another. While the task itself is simple, ratings are generally obtained for all possible pairs of sounds within the stimulus set, which often entails a large number of trials. Here, ratings were obtained for a curated set of sounds so as to facilitate the examination of auditory object and event perception both within and between the sound classes of vocal sounds (vowels from different human speakers), musical instruments, and human-environmental sounds (everyday objects and materials). The goal was to obtain a broader view of the acoustic features that undergird auditory object and event perception in humans than has been obtained previously.

Examining such a large variety of stimuli presents a number of non-trivial logistical and conceptual challenges. First, increasing the number of stimuli in a pairwise dissimilarity-rating task exponentially increases the number of trials and time required of participants (see Giordano et al., 2011, for discussion). Second, the relative importance or arbitrariness of some features, especially fundamental frequency, varies across categories. That is, fundamental frequency is likely a useful cue for the identification of some sound sources, such as for distinguishing speakers (Baumann and Belin, 2010), but it varies more arbitrarily when identifying others such as instruments, or can even be altogether absent or unreliable, such as for human-environmental sounds. We overcame these issues by using a set of careful experimental design choices informed by prior work on sound identification.

To address the issue of time demands, we relied on short duration sounds (250-ms, including the sounds’ onsets). While this may appear to be a quite restricted duration, findings from gating paradigms demonstrate that durations over 200-ms support asymptotic performance for many sound identification tasks (Gray, 1942; Robinson and Patterson, 1995a,b; Suied et al., 2014; Ogg et al., 2017), and this sort of controlled, limited duration has commonly been used in many neuroimaging studies (e.g., Formisano et al., 2008; Leaver and Rauschecker, 2010; Ogg et al., 2019). We focused on sound onsets because they have been shown to carry a large amount of information important for sound perception (Iverson and Krumhansl, 1993; Hjortkjær and McAdams, 2016) and identification (Saldanha and Corso, 1964; Wedin and Goude, 1972; Lemaitre and Heller, 2012; Ogg et al., 2019) and this choice naturally focuses our investigation on the critical timescales when real-world identification is taking place (i.e., after a sound has been initiated in the environment).

To address the differential importance of fundamental frequency across different sound types, we matched the fundamental frequency of each vowel utterance to instrument tokens by having instruments play the nearest corresponding note in the Western musical scale (or, for human-environmental tokens without a clear fundamental frequency, the instrument token played a note corresponding to the middle of the range of vowel fundamental frequencies). This allows fundamental frequency to vary naturally among auditory objects where it is likely a useful or identifiable cue (such as among speakers) while otherwise mitigating cross-category differences along this dimension (i.e., equating fundamental frequency among stimuli for which this is a more arbitrary cue). Human-environmental sounds were generally noise based and did not possess fundamental frequency information, so these sounds were not constrained in the same way. However, the degree of aperiodicity of the sounds was incorporated into the analyses to quantify the influence of this acoustic attribute.

Thus, we were able to control for a set of broad and potentially confounding acoustic differences between sound classes (fundamental frequency and duration), while allowing the natural acoustic features useful for sound identification within and between categories to vary. In doing so we were able to obtain a high level accounting of the acoustic dimensions that support auditory object perception.

Prior to describing the dissimilarity rating results, we first present the results of a control study on the identification of these sounds (Experiment 1). This preliminary study served as a check that the stimuli we chose as well as the durations and other acoustic controls we employed were representative of the sound sources we selected, and that our choice in duration did not distort the sound object or event that was conveyed by each token. Next, we present the results of a pairwise dissimilarity-rating study of the same stimuli (Experiment 2). This includes analyses of the acoustic features participants employed to distinguish these stimuli using both a typical MDS approach as well as an RSA-based approach. Finally, while identification and dissimilarity rating tasks are generally presumed to be inversely related, some results suggest interesting differences between these tasks that might inform our understanding of auditory object and event perception (Gygi et al., 2004, 2007; Siedenburg et al., 2016b). Thus, we conclude by comparing the identification and dissimilarity-rating data.

## Experiment 1: Sound Identification

### Materials and Methods

#### Participants

We recruited 24 participants (10 female) from the psychology department participant pool at the University of Maryland. Participants were compensated with course credit. Data from one participant were removed because they indicated they did not have normal hearing. Data from two additional participants were removed who reported possibly possessing perfect pitch. This was done for comparability with Experiment 2 and to mitigate a potential over-reliance on fundamental frequency cues among these listeners. The inclusion or removal of these participants did not alter the pattern of our results or conclusions. Participant ages ranged from 18 to 21 (*M* = 19.57, *SD* = 0.98). Participants were not selected based on their musical ability, but the majority (71%) had experienced some degree of musical training (*M* = 4.67 years, *SD* = 2.29), which is typical in a large university population (Schellenberg, 2006; Corrigall et al., 2013). The University of Maryland Institutional Review Board approved this study and all participants provided informed consent prior to participation.

#### Stimuli

We assembled a set of sound tokens representing 36 auditory objects and events that were evenly divided among three superordinate categories: (1) human vocal sounds (vowel utterances from different speakers), (2) musical instruments, and (3) sounds from everyday (human-environmental) objects and materials. These were high quality, natural sound tokens obtained from databases or via in-house recordings.

Vocal sound tokens were recorded in a sound treated room using a studio quality microphone, preamplifier and audio interface under conditions similar to Ogg et al. (2017). Three male and three female speakers (raised in the Mid-Atlantic region of the United States) read a randomized list of consonant-vowel-consonant utterances which comprised the crossing of the consonants /b/, /g/, and /h/ with the vowels /ɑ/ and /i/, all ending with the consonant /d/. One at least 250-ms excerpt of an /ɑ/ and /i/ was extracted for each speaker. The fundamental frequency of each utterance was then calculated using the YIN algorithm (De Cheveigné and Kawahara, 2002), and the median fundamental frequency of each vowel (12 total) was matched to the closest note in an equal-tempered scale: A_2_, A♭_*2*_, A♭_*3*_, B_2_, B_3_, B♭_*2*_, B♭_*3*_, C_3_, and G_2_ (range of raw fundamental frequency values: 99.16- to 251.38-Hz).

Musical instrument tokens were selected from the McGill University Master Samples database (Opolko and Wapnick, 1987). Instruments were chosen to sample the orchestral palette, and to match the fundamental frequency of the speakers’ utterances, which resulted in the use of the following instruments: acoustic guitar, piano, harp, contrabass (arco and pizzicato), cello (arco and pizzicato), bass clarinet, bassoon, french horn, trombone, and marimba. For each instrument we selected recordings from the database that matched the notes derived from the speaker utterances above, as well as E_3_ (the median of the range of vowel fundamental frequencies). Thus, we obtained 10 unique recordings of notes per instrument.

**FIGURE 1:**
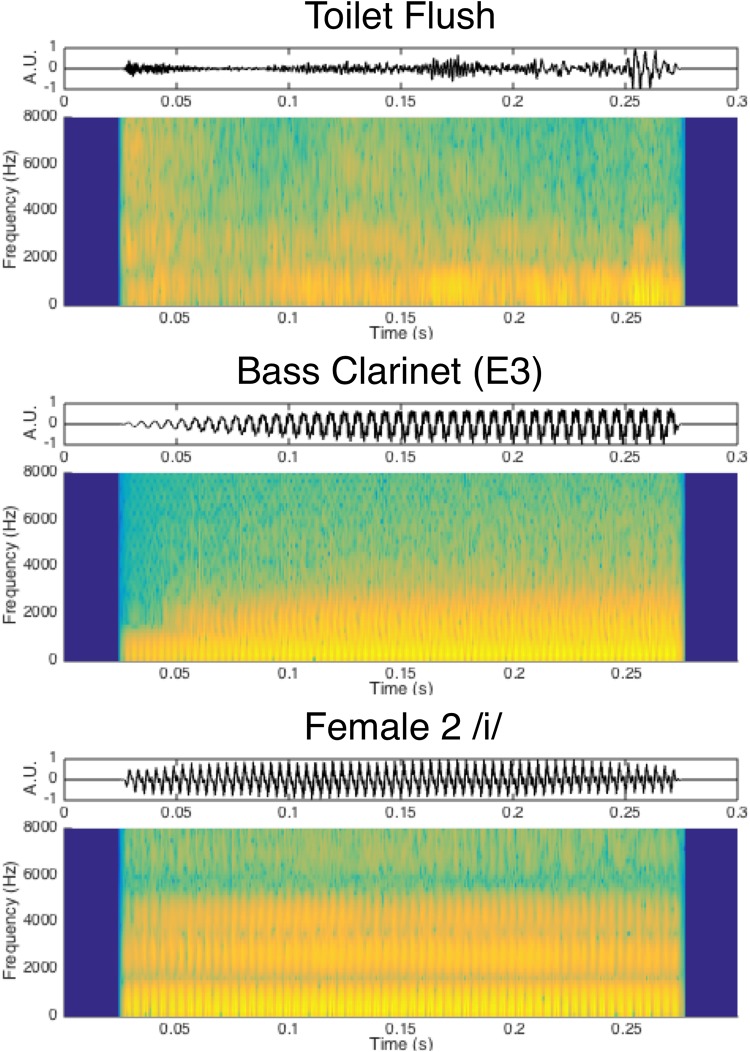
Waveform and spectrogram depictions of example sounds from each category.

**FIGURE 2:**
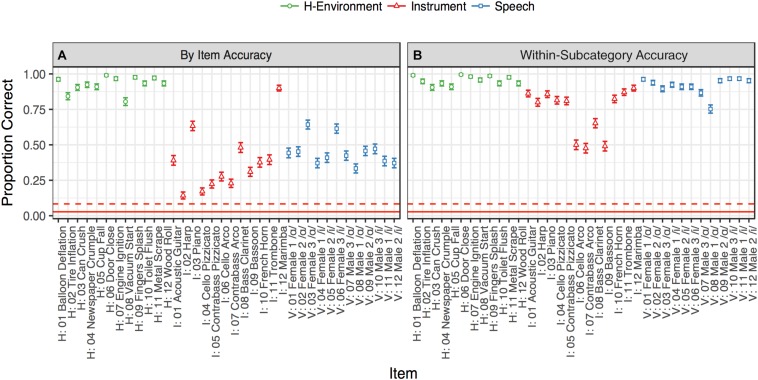
Identification accuracy rates in Experiment 1. The color and shape of the points denote sound category. The solid red line denotes 36-way chance performance (relative to all items: 1/36), and the dotted red line indicates within-category chance performance (relative to items in the same category for vocalizations, music, or human-environmental sounds: 1/12). Accuracy rates were aggregated for each item for each participant (across the 10 repetitions of each stimulus). Error bars denote ±1 standard error calculated across participants. Points in **(A)** are raw item-level accuracy rates while points in **(B)** code a response within a given subcategory (instrument family, vowel, or human-environmental media) as correct.

**FIGURE 3:**
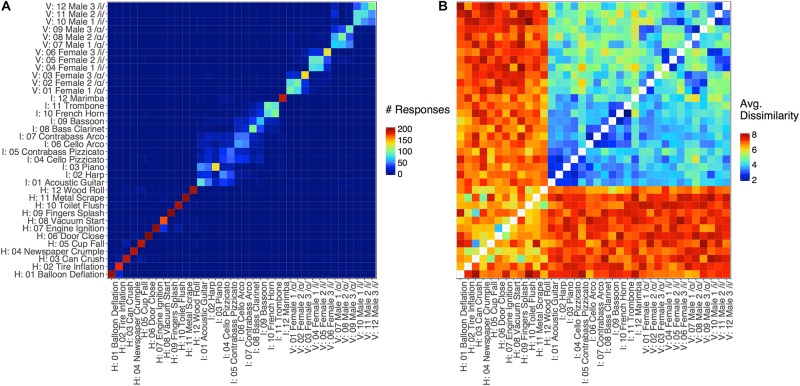
Dissimilarity matrices for **(A)** Experiment 1 (in which cells represent inter-item identification/confusion rates) and **(B)** Experiment 2 (in which cells represents dissimilarity ratings). Values for each item-pair entry in the matrix were aggregated (**A**: Experiment 1, summed; **B**: Experiment 2, averaged) over responses from all participants. The x-axis in **(A)** represents the item that was presented to the participant, and the y-axis indicates participants’ responses. The x- and y-axes in **(B)** indicate the first and second items that were presented, respectively. In both cases, stimulus labels are organized by category (vocal sounds, instrument sounds, and human environmental sounds). Note, the diagonal representing identical items pairs (comparing each item to itself) for the matrix in Experiment 2 was not presented to participants.

Human-environmental sounds comprised 12 everyday object sounds depicting a variety of media and events which were obtained from the BBC sound effect library (BBC, 1997, BBC Worldwide, London, United Kingdom) as well as the Carnegie Mellon University Sound Events and Real World Events databases (Carnegie Mellon University, 2008; Vettel, 2010). The selection of these sounds was inspired by the sound event taxonomy outlined by Gaver (1993) and examined by others (Lemaitre and Heller, 2013), with an emphasis on representing sounds typical of an everyday human environment. This included air and liquid sounds (2 tokens each) as well as sounds of complex interactions among solid materials (also 2 each): deformation, impact, mechanical, and movement sounds. These stimuli were predominantly noise-based and did not possess fundamental frequency information that could be accurately matched to the music and speech stimuli. This was confirmed by the YIN pitch detection algorithm, which returned highly variable fundamental frequency estimates for these tokens (IQR of fundamental frequency estimates for environmental tokens was 717 Hz on average, compared to 1.5 Hz among instrument and vocal sounds) and found these sounds to be largely noise-based (average aperiodicity of 0.25 among human-environmental sounds compared to 0.009 among music and vocal sounds). Because fundamental frequency was not a notable feature of these sounds, they were selected and presented without further constraints or controls along this acoustic dimension. Visual depictions of example sounds from each category are displayed in Figure 1. The full list of sounds can be found in Figures 2, 3.

Once the stimulus files from these categories were selected and gathered they were all pre-processed as follows. Stereo sounds were converted to mono by retaining only the left channel. Sounds were then edited to begin at their onset, defined here as the point at which the absolute value of the sound’s amplitude exceeded 10% of its maximum. Because the noise floor varied across stimuli, the onset point for each stimulus was determined based on a version of each sound file that was (4^th^ order, zero-phase Butterworth) high-pass filtered at 20 Hz. Each token was then trimmed to a duration of 250-ms from onset, and 5-ms onset and offset cosine ramps were applied. All stimuli were then normalized for RMS-level.

#### Apparatus

The sounds were first presented at approximately 50 dBA over Sennheiser HD 202-ii headphones (Sennheiser Electronics, GmBH, Wedemark, Germany), and participants were allowed to adjust the volume to a level they found comfortable during the introduction and practice sections. The experiment, playback and data collection were controlled via PsychoPy (version 1.82.01; Peirce, 2007) running on an Apple iMac (model number: MC508xx/A; Apple Computer, Cupertino, CA, United States) in a quiet, private room.

#### Procedure

Participants were instructed that on each trial they would hear a sound and their task was to simply push one of 36 buttons from a response key to indicate which sound they had heard. The label for each sound was matched to one of the 36 alpha-numeric computer buttons in the response key. The labels on the key that identified each stimulus were the same as in Figures 1, 2, except that for the speakers common, gender consistent first-names were used instead of the labels (e.g., “Male 1” changed to “Darren,” “Female 1” changed to “Annie” etc.). Participants were told they would always have access to the response key and that they did not need to explicitly memorize the response mappings. They were encouraged to take their time in selecting each response out of the 36 options and informed that response time was not being assessed in the interest of their responding as accurately as possible.

Participants were instructed that the sound set was made up of speech sounds (vowels) from various speakers, single notes from different musical instruments, or sounds of everyday objects, and that the pitch of the individual instrument sounds may vary to match the pitch of the vowel sounds. After reading the instructions, participants listened to a familiarization block wherein each sound was played while its label was displayed on the computer screen with no response required. Stimuli were played twice in the order they appeared on the response key (human-environmental first, followed by vowels and instruments played at E_3_), at a rate of one sound every 3 s. The label for each sound was displayed for 2 s, and 1 s prior to the next trial the on screen text cued the participant that the next sound was about to begin. Participants were told they could adjust the playback volume to a comfortable level during the familiarization and practice blocks, but not during the main study.

After the familiarization block came a practice block to introduce participants to the task. In the practice block, the stimuli were presented in a random order (instruments played at E_3_), and after each sound the participant was asked to indicate which sound they had heard by pressing the corresponding button from the response key. If they were unsure they were instructed to make their best guess. After responding, feedback was displayed on screen to indicate whether they were correct and, if not, which sound and response was the correct one. After each response, feedback was displayed for 2.5 s and then the next sound was presented.

The task in the main study was essentially the same as the practice block, but no feedback was provided. On each trial the response key schematic was displayed along with the prompt to press the keyboard button to indicate which sound they had heard (or to make their best guess if they were unsure). The sound for that trial was played 25-ms after the key and prompt appeared on screen. The prompt remained on screen until the subject made a response. After each response came a 1-s prompt indicating that the next trial was about to begin. The 36 sounds were presented over 10 blocks with no break or demarcation between blocks. The only change across blocks was the note of the instrument tokens, which played the same randomly selected note within each block. Thus each vowel and human-environmental token was presented 10 times, and each instrument timbre was presented 10 times at each of the 10 different notes. Sound order was randomized within each block and the order of blocks (instrument notes) was also randomized for each participant.

Participants also completed general demographics, language, musical experience, and handedness (Oldfield, 1971) questionnaires, as well as the Goldsmiths Musical Sophistication Index (Gold-MSI; Müllensiefen et al., 2014).

### Results

Accuracy rates for the identification of each stimulus are displayed in Figure 2, and a confusion matrix aggregating responses across participants is displayed in Figure 3A. The diagonal of the confusion matrix corresponds to item-level accuracy. We conducted one-tailed Wilcoxon signed-rank tests on the mean subject-level accuracies for each stimulus (proportion correct across the 10 trials of each item) to assess whether these items could be identified better than chance. These tests indicated that every item was identified reliably better than chance both across all stimuli (chance = 1/36; all FDR-corrected *p* < 0.05, Benjamini and Hochberg, 1995) and also among stimuli from within the same sound category (chance = 1/12; all FDR-corrected *p* < 0.05).

Although identification performance was above chance, accuracy rates differed widely by category. As shown in Figure 2A, human-environmental sound accuracy was near ceiling, followed by vocal sounds and then instrument accuracy. This observation was confirmed via a generalized binomial mixed-effect regression of trial-level accuracy using the “lme4” package (version 1.1-14, Bates et al., 2015) in R (version 3.3.3, R Core Team, 2017). This model contained a predictor for sound category (a factor with levels: vocal sounds, music or human-environmental; dummy coded with the human-environmental as the intercept), the musical training subscale of the Gold-MSI (z-normalized), and the interaction between these two variables. These models also contained random-effect intercepts for participants and each of the 36 items. This model indicated that instrument (*b* = −3.37, *z* = −10.16, *p* < 0.001) and vocal sound (*b* = −3.04, *z* = −9.20, *p* < 0.001) identification was less accurate than for human-environmental sounds, but that musical training bestowed a small performance advantage for these categories (interaction between musical training subscale and instrument: *b* = 0.15, *z* = 1.72, *p* = 0.09; and vocal sounds: *b* = 0.26, *z* = 3.01, *p* = 0.003).

Closer inspection of Figure 3A suggests a principled pattern of confusion among stimuli within each sound category. That is, participants typically confused similar sounds within subcategories of these three sound classes: instrument families (plucked strings: acoustic guitar, harp, piano, cello pizz., contrabass pizz.; bowed strings: cello arco, contrabass arco; woodwinds: bass clarinet, bassoon; brass: trombone, French horn;, or percussion: marimba), vowels (/ɑ/ or /i/ regardless of speaker), and human-environmental sound excitation media (air, deformation, impact, mechanical, liquid, and movement based sounds). To quantify this observation, we recoded accuracy to reflect within-subcategory accuracy rather than item-level accuracy. Specifically, any response that fell within a given subcategory (instrument family, vowel, or excitation media) was marked as correct, rather than the specific item itself. These within-subcategory accuracy rates, depicted in Figure 2B, were much higher, particularly for vowels (which were now near ceiling) and instruments.

The same binomial mixed-effect regression model described above was fit to this within-subcategory accuracy measure. Vocal sound accuracy in this model was no longer significantly different from human-environmental accuracy (*b* = −0.55, *z* = −1.59, *p* = 0.11). However, instrument accuracy was still significantly lower than human-environmental accuracy (*b* = −2.15, *z* = −6.40, *p* < 0.001), albeit less so than for the original, raw item-level accuracy. This was because woodwinds and bowed string instruments were frequently confused and correspond to different methods of excitation (see Figure 3A). This model also contained large positive interactions between musical training and both instrument (*b* = 0.40, *z* = 3.81, *p* < 0.001) and vocal sound (*b* = 0.78, *z* = 6.54, *p* < 0.001) accuracy suggesting that more musically trained participants did better on these stimulus types.

### Discussion

The results of this control study indicate that participants could reliably identify the stimuli they heard, and confused similar items within subcategories of vocal sounds (vowels) and instrument sounds (instrument families). In other words, while accuracy rates were not at ceiling for instrument and vowel stimuli, the confusions participants made were among conceptually similar stimuli, suggesting a set of common cues that participants relied on. Thus, these results indicate that the stimuli we selected are valid representations of these sounds despite the duration and fundamental frequency controls we employed.

The ostensibly low accuracy rates that we observed for instrument tokens are in fact quite similar to previously reported identification accuracy rates (Saldanha and Corso, 1964; Wedin and Goude, 1972). Our results are even consistent with this prior work in terms of some frequently confused and poorly identified stimuli such as the bassoon and cello (Wedin and Goude, 1972). Moreover, we note that the instrument stimulus set employed here is more diverse and nuanced than other work that obtained higher accuracy rates. Higher identification accuracy is usually observed in simpler stimulus sets that contain one representative of each instrument family (Robinson and Patterson, 1995a). For example, distinguishing among bowed strings, such as a cello and contrabass, or brass, such as a trombone and French horn, is non-trivial and is more difficult than distinguishing between those instrument families. More musically experienced listeners were able to better navigate these nuances and (unsurprisingly) performed better on instrument sounds. Perhaps less intuitively, however, more musically experienced participants also performed better when identifying vocalizations, which has also been noted in previous work (Chartrand and Belin, 2006).

For vocal sounds, listeners appeared to frequently confuse stimuli across speakers, while vowel identification was near ceiling. Again, these results are similar to prior work, which has found near ceiling vowel identification accuracy alongside high confusion rates among speakers (Tartter, 1991). This might have to do with the limited amount of training we chose to employ for comparability with human-environmental or instrument paradigms (which typically use very little training or familiarization; Ballas, 1993; Robinson and Patterson, 1995a). This contrasts with studies of speaker identification, which typically employ more extensive training phases with many sentences for individual speakers presented for familiarization (e.g., Perrachione and Wong, 2007). Note also that identifying the vocalizations in this study required recognizing both the speaker and the vowel that was being vocalized. On the one hand, this two-part identification requirement is a natural aspect of everyday speech perception (Creel and Bregman, 2011), but on the other hand this two-component process potentially poses more difficulty for the listener than the instrument and human-environmental sound identification trials. However, instead of this two-part task posing more difficulty overall, especially accurate vowel identification buoyed overall performance on vocalizations (compared to the instrument sounds, for example). Thus, participants relied on the easier aspect of the task and achieved reasonably good performance (despite frequently confusing across speakers).

Taken together the results of this preliminary study indicate that the stimulus controls we employed (particularly for duration and fundamental frequency) did not impair listeners’ ability to identify these sounds and the accuracy rates we observed for each class were similar to previous studies (Saldanha and Corso, 1964; Wedin and Goude, 1972; Tartter, 1991). We note that because these stimuli were not confused across the superordinate categories of music, vocal sounds and human-environmental sounds (or even very frequently outside of related subcategories within each class), this confusion matrix is very sparse, which makes acoustic analyses for these sounds somewhat difficult. To better assess the acoustic correlates of sound perception, we conducted a second experiment to obtain pairwise dissimilarity ratings among all possible pairs of these stimuli, yielding a richer matrix of perceived differences upon which to base a thorough acoustic analysis. That is, the results of Experiment 1 indicate that these sound tokens appear to be valid representations of the sounds we aimed to characterize and will thus be useful to examine further in a dissimilarity-rating task (Experiment 2) to probe the acoustic features participants use to differentiate these sounds. We will then return to these confusion rates in comparison to the pairwise rating data.

## Experiment 2: Pairwise Dissimilarity Ratings

### Materials and Methods

#### Participants

A new group of 54 participants (34 female) who did not participate in Experiment 1 were recruited from the psychology department participant pool at the University of Maryland to participate in Experiment 2. These participants were also compensated with course credit. Data from one participant were removed because they indicated they did not have normal hearing. Data from three additional participants were removed who reported possibly possessing perfect pitch. As in Experiment 1, the removal or inclusion of these participants did not alter the pattern of results or conclusions. Participant ages ranged from 18 to 29 (*M* = 20.06, *SD* = 2.06). Participants were not selected based on their musical ability, but as in Experiment 1, the majority (74%) had some degree of musical training (*M* = 4.88 years, *SD* = 3.65). The University of Maryland Institutional Review Board approved this study and all participants provided informed consent prior to participation.

#### Stimuli and Apparatus

The stimuli and apparatus for Experiment 2 were identical to Experiment 1.

#### Procedure

Participants first read the instructions for the study, which informed them that they would be rating how dissimilar pairs of sounds were on a scale from 1 (very similar) to 9 (very dissimilar) with the same general verbal description of the sounds as in Experiment 1. They were asked to use the full range of the rating scale and to keep their response criteria as consistent as possible throughout the study. As in Experiment 1, participants were told they could adjust the playback volume during the familiarization and practice blocks, but not during the main study.

In the familiarization block of Experiment 2 the 36 unique sounds (instruments played at E_3_) were played back in a randomized order with an inter-onset-interval of 1.6 s. Each trial began with a blank screen, and 25-ms of silence. After that initial 25-ms silent period a sound was played and, 25-ms after sound offset, a fixation cross was displayed for 1.3-s until the next trial began (no specific labels were provided).

Participants then practiced the rating task on a random subset of 15 stimulus pairs from the main study. Each trial began with the text “Sound 1” which remained on screen for 500-ms. 25-ms after this text appeared, the first sound in the pair for that trial was played. 500-ms after the “Sound 1” text disappeared, the same sequence occurred for “Sound 2” (thus a 1-s inter-onset-interval between the stimuli in each pair). After “Sound 2” had been displayed for 500-ms, the participant was prompted to press a button (1 through 9) to indicate how dissimilar the two sounds were. Listeners were only allowed to listen to each pair once. After the participant made their response, a 1-s text cue appeared on screen that indicated the next trial was about to begin.

The structure and timing of trials in the main study was identical to the practice block. The full pairwise crossing of our 36 stimuli (each speaker’s utterance, each instrument, and each human-environmental sound) yielded 1260 stimulus pairs (excluding the diagonal where each sound is paired with itself). This corresponds to the matrix in Figure 3B. In the complete set of 1260 possible pairs, each unique pair of stimuli would be presented twice in different orders (i.e., on either side of the diagonal in Figure 3: stimulus A then stimulus B, or stimulus B then stimulus A). In the interest of time and to minimize fatigue, each participant only rated one order of each unique pair and the order of pairs was counterbalanced across participants via two complementary lists of 630 pairs/trials, typical of many previous dissimilarity-rating studies (McAdams et al., 1995; Giordano et al., 2011; Siedenburg et al., 2016b). Each participant completed their 630 ratings over 3 blocks of 210 trials. Between each block, participants were given the option to take a short break, and they were reminded to keep their rating strategies as consistent as possible and to use the full rating scale in making their judgments.

On any trial where a vowel stimulus and an instrument stimulus were paired, the instrument token corresponding to the nearest equal-tempered note of the vowel’s fundamental frequency was played. Otherwise, instruments played the note E_3_ (the median of the range of vowel fundamental frequencies) when paired with other instruments or human-environmental sounds (which generally possessed little to no reliable fundamental frequency).

#### Data Analysis

Acoustic features for each stimulus were derived using packages in MATLAB (2014b, MathWorks, Natick, MA, United States). We analyzed features that have been previously shown to influence the perception of natural sound sources and events within or between the categories of speech, musical instrument, and human-environmental sounds. The literature pertaining to these features is described in the Section “The Acoustic Basis of Auditory Object and Event Perception Within Different Categories of Sound.” Table 1 provides a description of how each acoustic feature was calculated and used in the analysis along with notes regarding interpretation of the features and the software packages used. We obtained a single value to describe either a global stimulus feature (such as for attack-time), or measures of central tendency (median) and variability [inter-quartile range (IQR)] for temporally varying features that were calculated in windows throughout the stimuli (such as for spectral features). For features that were already inherent measures of variability over time (such as spectral variability and aperiodicity), only the median was retained.

**TABLE 1:** Description of acoustic features.

**Feature**	**Description**	**Interpretation**
Log-attack-time^1,g^	Log of the time difference between attack onset and ending	Lower values = faster onset time
Temporal centroid^1,g^	Center of gravity of the energy envelope	Lower values = earlier temporal centroid
Spectral centroid^2,m,i^	Center of gravity of the spectral (ERB) envelope	High value = higher frequency centroid
Spectral flatness^2,m,i^	Ratio of geometric and arithmetic means of the spectral (ERB) envelope	Measures noise/harmonic content. Higher values (1) are flatter/noisier
Spectral variability^2,m^	1 minus the correlation of ERB channel spectra between successive timepoints	Higher values = more variable
Aperiodicity^3,m^	Amount of aperiodic energy in the signal	Higher values = more aperiodic
ERB cochleagram^2,m,i,e^	Raw ERB cochleagram representation of each of 77 channels: 30 Hz – 16 kHz	Energy in each of channel over time
Modulation power spectrum^4,g,e^	2D-FFT of Gaussian spectrogram (50 dB dynamic range)	Maximum of 31.24 cyc/kHz and 47.92 Hz

*Derived via: ^1^Energy envelope or ^2^ERB cochleagram representation in the Timbre Toolbox (Peeters et al., 2011; Kazazis et al., 2017), ^3^YIN (De Cheveigné and Kawahara, 2002), and ^4^modulation power spectrum (Elliott and Theunissen, 2009). Feature calculated to reflect a ^*g*^global quality of the stimulus or the ^*m*^median or ^*i*^IQR of temporally varying features. Some features were used only in the pairwise correlation analyses (i.e., not in the MDS analysis) based on the ^*e*^Euclidean distance of multi-value representations.*

The reliability of dissimilarity ratings across participants was assessed via split-half correlations that were adjusted by the Spearman–Brown prophecy formula using the “multicon” (version 1.6, Sherman, 2015) package in R. We then performed two complimentary acoustic analyses in the style of both MDS and RSA.

First, we conducted an ordinal MDS analysis of the group-level dissimilarity matrix (shown in Figure 3B) using the “smacof” package (version 1.6-6, de Leeuw and Mair, 2009) in R. For this, the diagonal of the dissimilarity matrix (corresponding to identical stimulus pairs, which we did not present) was defined as the minimum possible response value (1, or the lowest dissimilarity). The positions of the stimuli along each dimension were then correlated with their acoustic features values using Kendall’s tau correlations (using features associated with the E_3_ musical instrument stimuli, although similar results were obtained using features averaged across notes), and the set of correlations with each dimension were false-discovery rate corrected.

**FIGURE 4:**
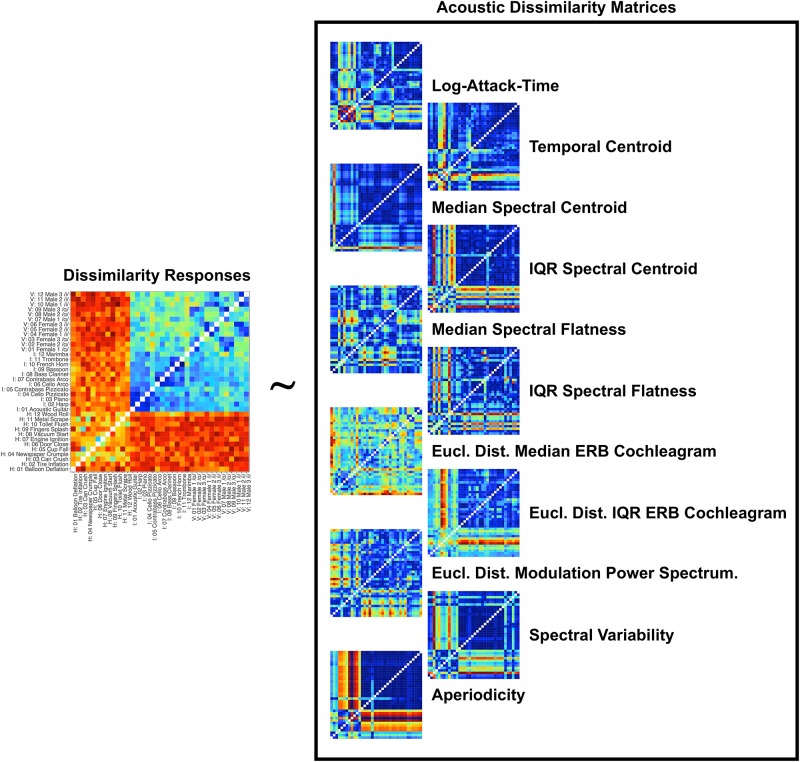
Schematic of the RSA analysis and depictions of the item-pair differences for each acoustic feature in the matrix. Group-averaged dissimilarity ratings for each stimulus pair (from Figure 3B) were correlated with how the pairs differ in terms of different acoustic feature dimensions. Differences between the items for each acoustic feature were calculated by taking the absolute value of the difference for that feature between each pair of stimuli. Item-pair differences for multi-value representations (such as modulation power spectra and ERB cochleagram representations) were calculated by taking the Euclidean distance between the values in the representations of each item pair.

**FIGURE 5:**
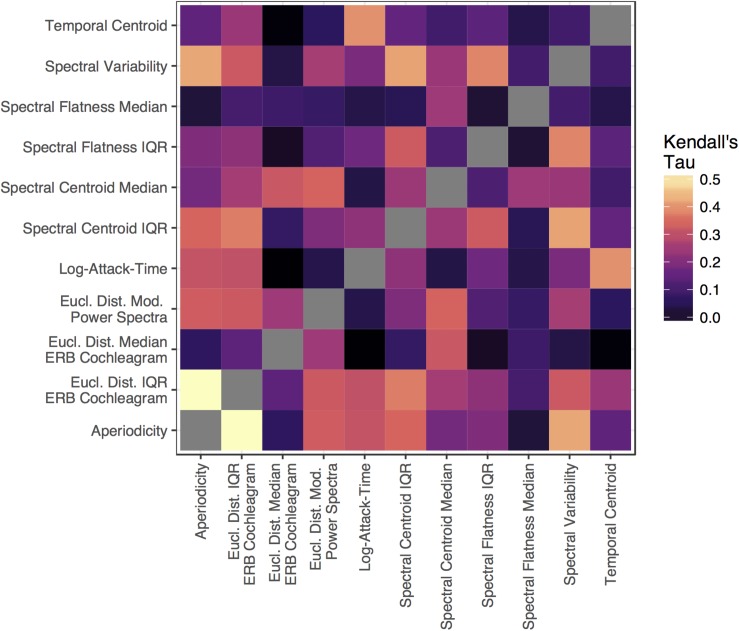
The (Kendall’s tau, rank-ordered) correlation between each acoustic feature matrix depicted in Figure 4.

The RSA analysis examined correlations between group-averaged item-pair-level dissimilarity ratings and the acoustic feature differences of each pair. This amounts to a correlation between the group-averaged dissimilarity rating matrix in Figure 3B and the corresponding differences between each pair of sounds in terms of each acoustic feature (absolute value of the difference for each feature between the two sounds on each trial, or Euclidean distances for multi-value representations; see Table 1). This analysis and the dissimilarity matrices of each acoustic feature are depicted in Figure 4. The relationships (correlations) among the acoustic dissimilarity matrices are depicted in Figure 5.

We calculated both standard rank-ordered Kendall’s tau correlations for each feature (i.e., between item-pair-wise dissimilarity ratings and the item-pair-wise differences for each acoustic feature) as well as semi-partial Kendall’s tau correlations for each feature while holding the other features constant. Statistical significance for each correlation was assessed using non-parametric bootstrapped methods, where the correspondence between the dissimilarity ratings and the acoustic features of each item pair were randomly shuffled before calculating the correlation statistic over many iterations (*n* = 10,000 iterations) to create a null distribution. The number of times the shuffled correlation statistics met or exceeded the observed statistics for the non-shuffled data was recorded (*r*) and used to generate a *p*-value for each correlation [as in North et al., 2002: (*r*+ 1)/(*n* + 1)].

These correlation analyses were then repeated for specific subsets of the item-pairs to assess the influence of these acoustic features on specific within or between category dissimilarity ratings. Fundamental frequency-based features were included to quantify the influence of this acoustic quality among comparisons where this feature could be informative (i.e., allowed to vary; specifically, F0 median and IQR were included in the vocal vs. vocal analysis and F0 IQR was included in the vocal vs. instrument analysis).

The Supplementary Material describes a similar approach using an all-subsets-regression (model comparison) analysis with linear-mixed effects models to identify the most parsimonious set of acoustic features that could predict trial-level dissimilarity ratings as a function of differences along each acoustic feature dimension above. This analysis revealed similar results to the correlation analyses. The influence of musical training on these models was also assessed in a subsequent all-subsets-regression that included the interaction between the best fitting features and participants’ musical sophistication.

### Results

Average dissimilarity ratings for each stimulus pair (averaged across participants) are displayed in Figure 3B. Participants were very reliable (0.98 split-half correlations adjusted by the Spearman–Brown prophecy formula) in their dissimilarity responses across item pairs, which is similar to the high reliability observed in previous work (Wedin and Goude, 1972). Thus, we proceeded with analyses to better understand what acoustic features participants used to make their dissimilarity judgments.

#### Multidimensional Scaling

**FIGURE 6:**
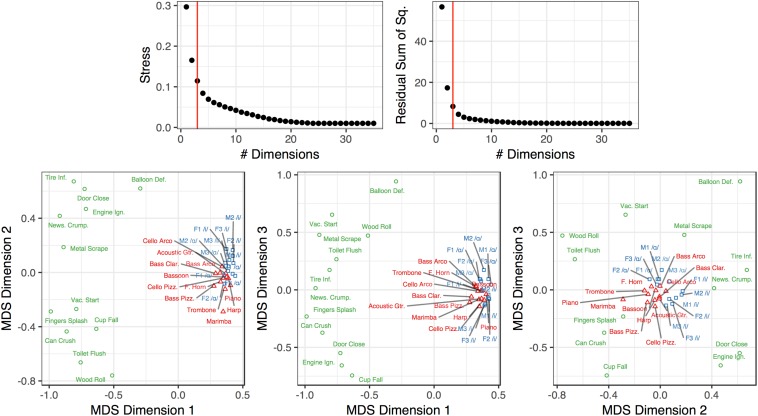
Multidimensional scaling analysis of the dissimilarity ratings from Experiment 2. The red vertical bar in the top graphs indicates the selected number of dimensions in the plots below. The color and shape of the points and labels denote the sound’s category. Dimension 1 corresponds to spectral variability/aperiodicity. Dimension 2 corresponds to the spectral envelope. Dimension 3 corresponds to the temporal envelope (see section “Multidimensional Scaling”).

Figure 6 depicts the MDS analysis we conducted on the group-averaged dissimilarity matrix (shown in Figure 3B). Three dimensions provided good fit and stress values compared to other dimensional solutions. We also ran a four-dimensional solution but this proved sub-optimal because the 4^th^ dimension was difficult to interpret conceptually or acoustically (no significant correlation with acoustic features), and did not yield a substantial improvement in fit over a three-dimensional solution. To understand how the coordinates of the stimuli in this three-dimensional space related to their acoustic properties, we correlated the acoustic feature values of the stimuli (see Table 1) with their positions along each dimension. This revealed that Dimension 1 related to spectral variability and aperiodicity (top four correlations: aperiodicity: *r*_τ_ = −0.42; spectral centroid IQR: *r*_τ_ = −0.40; spectral flatness IQR: −0.36; spectral variability: *r*_τ_ = −0.31; all FDR-corrected *p* < 0.05), Dimension 2 related to the spectral envelope (spectral centroid median: *r*_τ_ = −0.36; spectral flatness median: *r*_τ_ = −0.32, all FDR-corrected *p* < 0.05), and Dimension 3 related to the temporal envelope (temporal centroid: *r*_τ_ = −0.45; log-attack-time: *r*_τ_ = −0.40, all FDR-corrected *p* < 0.05). We note that both spectral variability and aperiodicity were associated with Dimension 1. This is likely because of how conceptually similar these features are (and therefore are likely to be related to one another): any aperiodic signal is going to also be uncorrelated with itself from moment to moment, which results in a higher spectral variability value.

#### Representational Similarity Analysis

Multidimensional scaling analyses are essentially dimensionality reduction techniques, which fit dissimilarity data to a low dimensional space that can be intuitively visualized and interpreted. By the same token, such low-dimensional solutions are constrained by visual interpretability and thus are typically limited to two or three dimensions. This low number of dimensions might overlook interesting variance that exists in the data. The large number of stimuli we used as well as the diversity among them resulted in a rich pattern of dissimilarity responses (see Figure 3B). Thus, we wondered if large, overriding acoustic differences might dominate a low-dimensional solution, and obscure additional, interesting results which could be uncovered by a technique that was able to leverage more variability in the data. To do this, we correlated group-averaged dissimilarity rates among the pairs of stimuli with how each pair differed along a set of acoustic dimensions (see Figure 4 and Table 1). In addition to standard (rank-ordered) correlation statistics, we also calculated semi-partial correlations to isolate the variance accounted for by each individual feature (i.e., while holding the other features constant).

**FIGURE 7:**
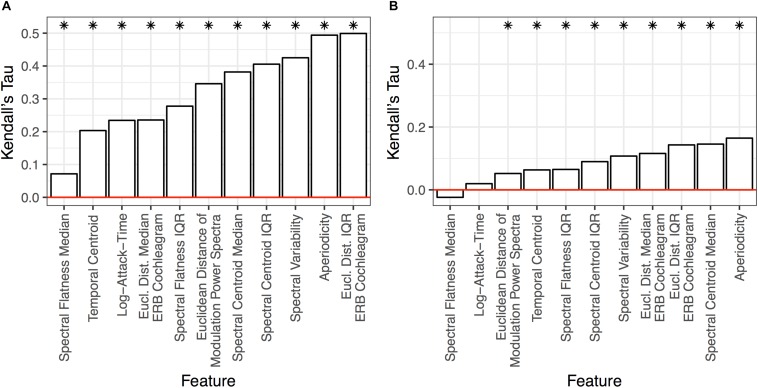
Results of the standard **(A)** and semi-partial **(B)** correlation analyses over all item-pairs. Features are ranked from the lowest (left) to highest (right) correlation along the x-axis. Asterisks indicate features that are significant (*p* < 0.05) based on non-parametric bootstrap analyses that shuffled the association between dissimilarity responses and each item pair (see section “Data Analysis”) in the group-level dissimilarity matrix (Figure 3B). Positive correlations indicate that larger differences among stimulus pairs along that dimension are associated with larger dissimilarity responses.

**FIGURE 8:**
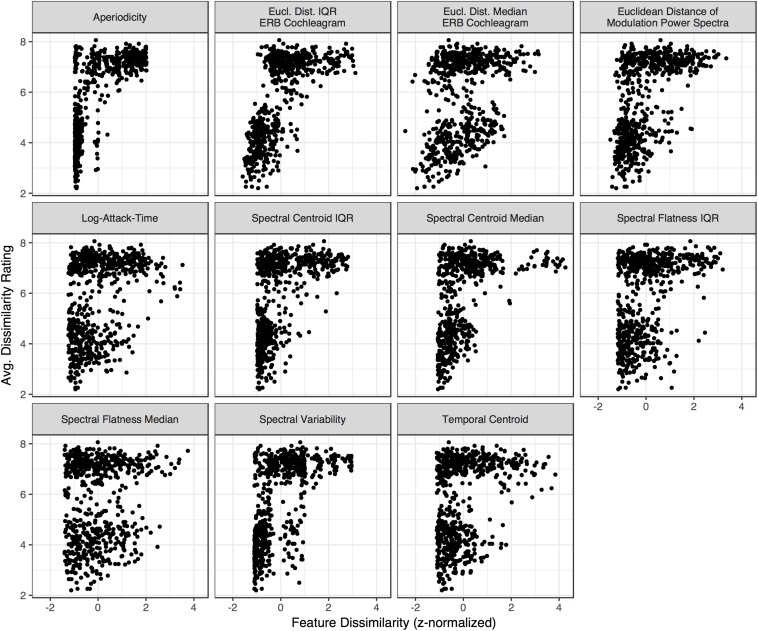
Dissimilarity ratings for each stimulus pair in the group-averaged matrix (Figure 3B) plotted against how each pair differs along each acoustic dimension (Figure 4). Differences are centered and z-normalized within each acoustic feature (x-axis).

The results of these analyses are summarized in Figures 7, 8 and Tables 2, 3. Standard and semi-partial correlations both indicated a significant association between dissimilarity responses and aperiodicity, spectral variability (overall, and based on the IQR of the spectral centroid and ERB cochleagram channels) as well as the spectral envelope (spectral centroid and the median of ERB cochleagram channels). The sounds’ temporal envelopes (temporal centroid) were also associated with dissimilarity responses, albeit to a lesser degree than the other features above. The standard correlations were generally higher (cf. Figures 7A,B), and suggested a larger influence of different spectral variability features. However, when the collinearity among these features was accounted for in the semi-partial correlations, these associations (particularly among many variability-based features) were more modest.

**TABLE 2:** RSA correlation analysis results: standard Kendall’s tau.

		**Within-category**			**Between-category**		
	**Overall**	**H-environment v.s. H-environment**	**Instrument v.s. Instrument**	**Vocalization v.s. Vocalization**	**H-environment v.s. Instrument**	**H-environment v.s. Vocalization**	**Vocalization v.s. Instrument**
Aperiodicity	0.49(0.001)*⁣**	0.17(0.021)*	0.2(0.01)*	0.09 (0.135)	−0.02(0.601)	0.07 (0.097)	0.12(0.015)*
Euclidean distance IQR ERB cochleagram	0.5(0.001)*⁣**	0.1 (0.118)	0.25(0.002)**	0.4(0.001)*⁣**	0.06 (0.155)	0.1(0.036)*	0.24(0.001)*⁣**
Euclidean distance median ERB cochleagram	0.24(0.001)*⁣**	0.34(0.001)*⁣**	0.35(0.001)*⁣**	0.52(0.001)*⁣**	0.21(0.001)*⁣**	−0.06(0.868)	0.2(0.001)*⁣**
Euclidean distance: modulation power spectra	0.35(0.001)*⁣**	0.14(0.05)*	0.24(0.002)**	0.13 (0.062)	0.16(0.002)**	−0.01(0.571)	0.09 (0.057)
Log-attack-time	0.23(0.001)*⁣**	−0.02(0.58)	0.31(0.001)*⁣**	0.01 (0.467)	0.04 (0.23)	0.07 (0.113)	−0.07(0.873)
Spectral centroid IQR	0.41(0.001)*⁣**	−0.1(0.887)	0.22(0.004)**	0.06 (0.239)	0.05 (0.176)	0.11(0.029)*	0.05 (0.202)
Spectral centroid median	0.38(0.001)*⁣**	0.15(0.035)*	0.04 (0.328)	0.48(0.001)*⁣**	0.22(0.001)*⁣**	−0.05(0.807)	0.13(0.011)*
Spectral flatness IQR	0.28(0.001)*⁣**	0.25(0.001)**	0.1 (0.14)	0 (0.522)	0 (0.473)	0.09 (0.061)	0.07 (0.131)
Spectral flatness median	0.07(0.005)**	0 (0.492)	0.09 (0.149)	0.11 (0.095)	0.12(0.017)*	0.01 (0.452)	0.06 (0.143)
Spectral variability	0.43(0.001)*⁣**	0.26(0.001)*⁣**	0.15(0.041)*	0.08 (0.176)	0 (0.502)	0.04 (0.248)	0.2(0.001)*⁣**
Temporal centroid	0.2(0.001)*⁣**	0.05 (0.273)	0.32(0.001)*⁣**	0.08 (0.158)	0.08 (0.084)	0.13(0.01)*	0.08 (0.089)
F0 IQR	NA	NA	NA	−0.1(0.885)	NA	NA	0.11(0.024)*
F0 median	NA	NA	NA	0.13 (0.063)	NA	NA	NA

^*^p < 0.05; ^∗∗^*p* < 0.01; ^∗∗∗^*p* < 0.001.

**TABLE 3:** RSA correlation analysis results: semi-partial Kendall’s tau.

		**Within-category**			**Between-category**		
	**Overall**	**H-environment v.s. H-environment**	**Instrument v.s. Instrument**	**Vocalization v.s. Vocalization**	**H-environment v.s. Instrument**	**H-environment v.s. Vocalization**	**Vocalization v.s. Instrument**
Aperiodicity	0.16(0.001)*⁣**	0.07 (0.171)	0.09 (0.07)	0 (0.5)	0.02 (0.344)	0.05 (0.125)	0.06 (0.126)
Euclidean distance IQR ERB cochleagram	0.14(0.001)*⁣**	0.14(0.04)*	0.07 (0.188)	0.17(0.001)**	0.02 (0.324)	0.06 (0.122)	0.14(0.003)**
Euclidean distance median ERB cochleagram	0.12(0.001)*⁣**	0.28(0.001)*⁣**	0.29(0.001)*⁣**	0.24(0.001)*⁣**	0.13(0.004)**	−0.01(0.537)	0.18(0.001)*⁣**
Euclidean distance: modulation power spectra	0.05(0.015)*	0 (0.482)	0.08 (0.148)	0.03 (0.343)	0.09(0.023)*	0.01 (0.44)	0.01 (0.421)
Log-attack-time	0.02 (0.194)	−0.02(0.621)	0.13(0.021)*	−0.03(0.685)	0.07 (0.084)	−0.01(0.578)	−0.09(0.971)
Spectral centroid IQR	0.09(0.001)*⁣**	−0.08(0.827)	0.2(0.004)**	−0.04(0.7)	0.02 (0.366)	0.08 (0.071)	−0.03(0.692)
Spectral centroid median	0.15(0.001)*⁣**	0.03 (0.341)	0 (0.487)	0.25(0.001)*⁣**	0.12(0.002)**	0 (0.495)	0.06 (0.085)
Spectral flatness IQR	0.06(0.005)**	0.15(0.02)*	0.04 (0.301)	0.02 (0.402)	0.04 (0.187)	0.06 (0.082)	0.02 (0.391)
Spectral flatness median	−0.02(0.828)	0 (0.485)	0.12 (0.07)	0.02 (0.382)	0.05 (0.175)	0 (0.499)	0.01 (0.438)
Spectral variability	0.11(0.001)*⁣**	0.14(0.024)*	−0.01(0.579)	0.02 (0.378)	0.06 (0.112)	0 (0.535)	0.15(0.001)*⁣**
Temporal centroid	0.06(0.005)**	0.04 (0.281)	0.16(0.006)**	0.05 (0.216)	0.07 (0.079)	0.12(0.011)*	0.09(0.032)*
F0 IQR	NA	NA	NA	−0.05(0.745)	NA	NA	0.1(0.032)*
F0 median	NA	NA	NA	0.05 (0.195)	NA	NA	NA

^*^p < 0.05; ^∗∗^*p* < 0.01; ^∗∗∗^*p* < 0.001.

Similar features were implicated among the top-ranked models in an all-subsets-regression analysis using linear mixed effects models of trial-level data (see Supplementary Material). Including musical training and its interaction with the top-ranked acoustic features improved model fit: a higher degree of musical training (which did not significantly influence responses on its own) was found to temper the influence of aperiodicity, and spectral centroid via negative interactions. This suggests that more musically experienced participants relied less on these acoustic cues when making dissimilarity judgments.

#### Representational Similarity Analysis for Specific Between and Within Category Dissimilarity Judgments

One strength of the design of our dissimilarity rating study and the controls we implemented among our stimuli is that we were able to obtain dissimilarity ratings among sounds both within and between specific sound categories. Not only does this effectively amount to three comparable dissimilarity rating studies for each individual sound category (i.e., based on responses from the same participants, with the same set of stimuli) but this approach also facilitates between-category comparisons. This allows us to determine the relative importance of these acoustic cues within and across different sound categories, and to examine how those features are used to make category-level distinctions between different sound types (e.g., vocal vs. instrument sounds). Such a comparable level of analysis within and between categories has not been carried out in this way in prior work. Thus, we repeated the correlational RSA analysis above based on specific subsets of item pairs within the group-level dissimilarity matrix shown in Figure 3B.

**FIGURE 9:**
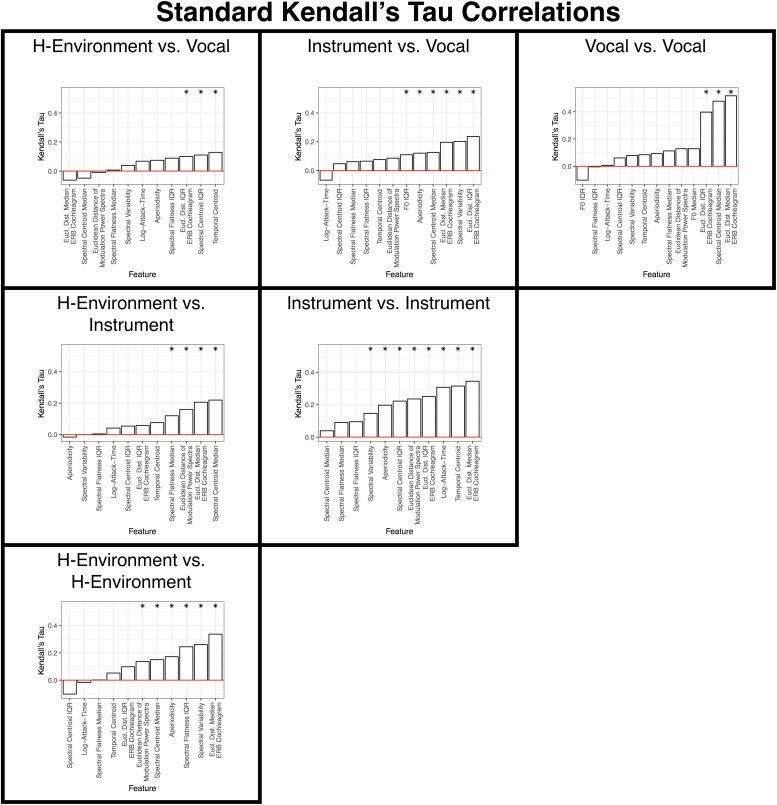
Results of the standard correlation analyses for each within- and between-category subsection of the group-level dissimilarity rating matrix. Each panel is labeled according to the sound pairs that were analyzed and panels are organized to align with where these categories intersect in the group-level dissimilarity rating matrix shown in Figure 3B. Features are ranked from the lowest (left) to highest (right) correlation along the x-axis. Asterisks indicate features that are significant (*p* < 0.05) based on non-parametric bootstrap analyses that shuffled the association between dissimilarity responses and each item pair (see section “Data Analysis”) in the group-level dissimilarity matrix (Figure 3B). Positive correlations indicate that larger differences among stimulus pairs along that dimension are associated with larger dissimilarity responses. See also Table 2.

**FIGURE 10:**
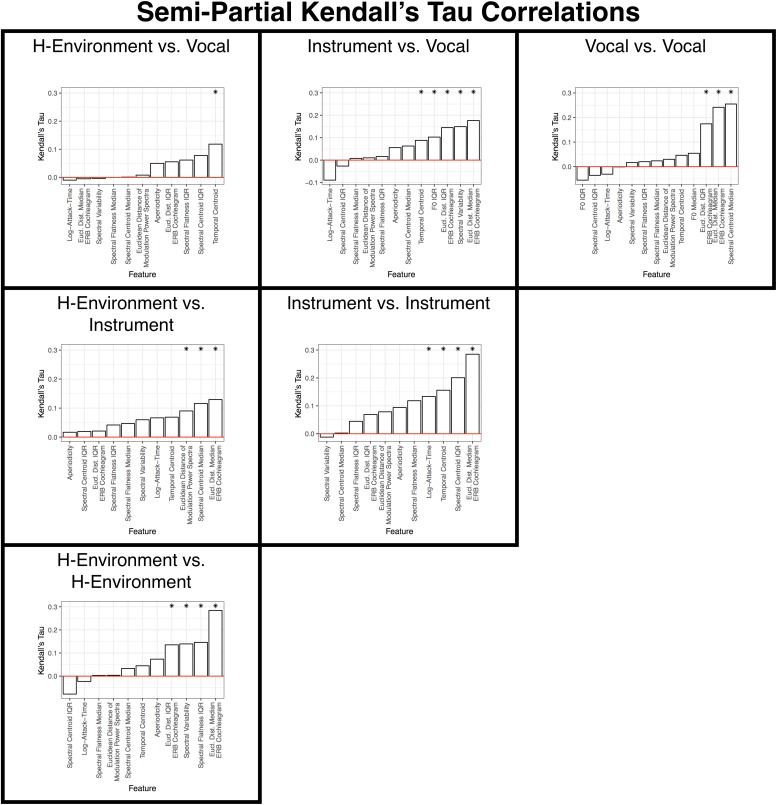
Results of the semi-partial correlation analyses for each within- and between-category subsection of the group-level dissimilarity rating matrix. Each panel is labeled according to the sound pairs that were analyzed, and panels are organized to align with where these categories intersect in the group-level dissimilarity rating matrix shown in Figure 3B. Features are ranked from the lowest (left) to highest (right) correlation along the x-axis. Asterisks indicate features that are significant (*p* < 0.05) based on non-parametric bootstrap analyses that shuffled the association between dissimilarity responses and each item pair (see section “Data Analysis”) in the group-level dissimilarity matrix (Figure 3B). Positive correlations indicate that larger differences among stimulus pairs along that dimension are associated with larger dissimilarity responses. See also Table 3.

These results are summarized in Figures 9, 10 and Tables 2, 3. Spectral envelopes generally played the most prominent role across comparisons, although temporal envelopes (temporal centroid) were associated with dissimilarity judgments between the vocalizations (which were mostly steady-state vowels in this case) and human-environmental sounds (more variable temporal envelopes), and for distinguishing instruments from one another. Spectral variability measures were also associated with distinguishing most categories from one another. Fundamental frequency variability was associated with how participants distinguished instruments from vocalizations. Finally, aperiodicity was noticeably absent from these analyses among subcategories. Thus, this feature appears to play a higher-level role distinguishing the predominantly noisy human-environmental sounds from instrument and speech sounds, which have stronger fundamental frequencies and regular harmonic structures.

#### Comparing Identification (Confusion) Responses and Dissimilarity Ratings

Identification (or confusion) data and direct dissimilarity ratings both yield pairwise matrices of differences among stimuli (Figure 3). Intuitively, the more dissimilar two items are, the less likely they are to be confused. We conducted the identification study in Experiment 1 primarily to make sure our sound tokens and acoustic controls yielded good representations of the sounds we were trying to characterize. However, there is some question as to how related identification and dissimilarity rating tasks are from a psychological and computational perspective (Giordano and McAdams, 2010; Siedenburg et al., 2016b), so comparing Experiments 1 and 2 might shed additional light on this issue. Indeed, while the matrices from these experiments appear to tell a similar story, there are important differences that need to be kept in mind.

First, the confusion data are very sparse relative to the dissimilarity rating data. For example, there was very little confusion among stimuli across categories in Experiment 1. At the same time, accuracy for the human-environmental sounds was very high, which gives an analysis of confusion responses for these stimuli very little variability in the outcome measure to predict. Second, the diagonal of an identification confusion matrix is informative, because it pertains to item-level accuracy. However, the diagonal is not typically analyzed or obtained for dissimilarity rating data. This leads to the final difference between these data, which is that the entries in a confusion matrix are zero-sum: a confusion of one stimulus for another means that there is no confusion for a different stimulus on that trial (or increased accuracy if the response is correct). Meanwhile, the entries in a pairwise dissimilarity-rating matrix are more independent and comprehensive (simple 1 to 9 ratings for each pair).

Despite these core differences and corresponding notes of caution, it is nonetheless informative to explore any correspondence between the identification (confusion) results and the dissimilarity ratings given that few studies have directly compared such datasets. Thus, we correlated the confusion (Figure 3A) and dissimilarity rating matrices (Figure 3B). For this, we averaged the entries for the item pairs in each matrix across their respective diagonals. This is because it is not clear how stimulus and response in the identification task maps onto the item order in a pairwise rating task. Additionally, the diagonal in the dissimilarity rating data was assigned the minimum possible response value (1). The correlation between these matrices yielded a significant, although moderately sized, Kendall’s tau correlation (*r*_τ_ = −0.49, *p* < 0.001). A similar correlation (*r*_τ_ = −0.42, *p* < 0.001) was obtained based only on item pairs present in both matrices (i.e., excluding the diagonal and cells where there were no confusions). Thus, while the correlation between these two datasets is significant, their association is rather modest.

### Discussion

We obtained dissimilarity ratings from a large number of subjects for a diverse set of auditory objects and events that encompassed many sounds that are important in everyday human life. Studying these diverse natural sounds together helps to synthesize bodies of literature on individual sound categories that have until now mostly been studied in isolation. This in turn has precluded a broader view of the general acoustic dimensions that guide a listener’s perception of auditory objects and events. We found that a sound’s aperiodicity, spectral variability, spectral envelope, and temporal envelope play a strong role in how participants distinguish different natural auditory stimuli. Moreover, the usefulness of these features varied for certain within or between category comparisons. These represent core acoustic dimensions that have been extensively studied in psychophysical (Moore, 2012), computational (Peeters et al., 2011), and neuroscientific (Giordano et al., 2011) areas. However, our results go further to demonstrate that these features also relate to how listeners distinguish natural auditory objects and events across a wide range of human-relevant sounds.

We show that acoustic features identified in previous, within-category studies generalize to other kinds of auditory objects and events. Timbre, for example, is a particularly well-studied class of sound perception and has been shown to relate to spectral centroid (spectral envelope) and log-attack-time (temporal envelope; Iverson and Krumhansl, 1993; McAdams et al., 1995). Similar features (albeit swapping log-attack-time for temporal centroid) have been associated with environmental object and event perception (Hjortkjær and McAdams, 2016). Spectral variability has been only intermittently identified among the set of features that influence timbre perception (Caclin et al., 2005) but has been more reliably identified in studies of environmental sounds (Gygi et al., 2004, 2007). Our results largely confirm these results, both at a higher level (when comparing instrument, vocalization, and human-environmental sounds all together) and in specific within and between category comparisons. In line with the environmental sound literature, our data suggest that spectrotemporal variability along with aperiodicity plays a prominent role in auditory object and event perception that perhaps is not fully engaged by the comparative regularity of instrument spectra compared to speech or other human-environmental sounds (Huang and Elhilali, 2017; Ogg et al., 2017).

Additionally, by studying multiple sound categories together, we have identified features that are useful for distinguishing between them. For example, both spectral envelope and spectral variability cues were useful when comparing all three sound categories as well as in most specific within and between category analyses. In addition to spectral variability, our data highlight the importance of aperiodicity in distinguishing among different classes of sounds, similar to previous work comparing different sound categories (Huang and Elhilali, 2017; Ogg et al., 2017). Fundamental frequency variability has also been previously noted to be useful for listeners when distinguishing speech and instrument sounds (Ogg et al., 2017), which is a finding we replicate here. Taken together, these results suggest that we can expand the groundwork and dimensional space outlined by research on musical timbre so as to include dimensions relating to aperiodicity and spectral variability. This would then allow us to explain how the diverse array of stimuli and sound categories examined here relate to one another.

Our results also touch on questions regarding the relationship between semantic and perceptual or acoustic knowledge (Siedenburg et al., 2016b). Our identification and confusion data were significantly correlated with our dissimilarity rating data but the correlation was modest. Such an incomplete alignment between identification (which relies more explicitly on semantic knowledge) and dissimilarity rating tasks (which are more perceptual or feature based) has been noted previously (Giordano et al., 2010; Siedenburg et al., 2016b). Additionally, higher levels of musical training in the dissimilarity task served only to decrease the influence of acoustic features. This result comes despite the increased identification accuracy we observed for music and vowel sounds among more musically trained individuals. Taken together, these results suggest that identification and dissimilarity rating tasks may tap non-overlapping psychological processes that can potentially be modified by training. Our results suggest that training might lead to a decreased reliance on physical stimulus properties and an increased reliance on potentially non-acoustic (and likely semantic) cues. Of course, this is speculative and emphasizes the need for further work to better describe the influence of acoustic and semantic knowledge in performance on these tasks.

We note a few limitations in our work that could be further investigated by follow up studies. First, our identification (confusion) matrix was very sparse and we observed especially high accuracy rates for the human-environmental stimuli. This ceiling effect (both for the human-environment stimuli overall and the minimal between-category confusions) complicated an acoustic analysis of these data. Indeed, in preliminary analyses, MDS solutions for the identification data were very difficult to interpret, likely because of the structure and sparsity of this matrix. A more thorough probe of acoustic influences on identification performance might be better realized through follow up work that directly manipulates specific acoustic qualities, such as those indicated by Experiment 2 and by other work (Gygi et al., 2004; Thoret et al., 2016; Venezia et al., 2016). The sparsity of the identification data also limits the comparison between identification and dissimilarity matrices, as does the fact that different participants performed these two tasks. It is worth pointing out that the identification task used here was mainly a control task, designed to check the validity of our stimuli and controls, and that the aim of the current work was squarely at deepening our understanding of acoustic processes as assessed by the dissimilarity rating task. Thus, further questions regarding semantic knowledge and its relationship with identification and dissimilarity rating tasks might be best served by follow-up work, ideally comparing data from the same participants in both tasks. Finally, we obtained a participant sample that contained a wide range of musical training, but these participants were predominantly not college trained music students or professionals. Thus, while our results suggest an influence of musical training, a deeper analysis of this issue would be well-served by assessing participants across a wider spectrum of musical training and experience.

Our results help connect different areas of research on natural sound perception by facilitating a comparable analysis of how listeners distinguish sounds within and between these categories. The data we provide here are useful for generating new hypotheses and for identifying more general perceptual processes that are at play during auditory object recognition. We relied on classic techniques in the field to examine ideas that have been outlined previously (see McAdams, 1993; Handel, 1995 for similar discussions). However, an important limitation of these techniques is that they do not reveal what variance is accounted for by acoustic properties and what is accounted for by a listener’s semantic differentiation among sounds. We would therefore like to amplify calls made by others (e.g., Giordano et al., 2010; Siedenburg et al., 2016a,b; Siedenburg and McAdams, 2017) for new techniques and approaches to disentangling these issues in object recognition. The present study aimed to broaden the scope of this issue by connecting important auditory perception literatures, but follow-up work could benefit the field by developing novel tasks and approaches. Some potential avenues include (1) manipulating or morphing stimuli across feature dimensions or between categories, (2) creating more difficult attention demands toward different acoustic features or categories within tasks, or (3) constraining analyses of behavioral responses based on neural responses.

## Conclusion

Auditory object and event perception is a core facet of the auditory system that cuts across many domains of research including the perception of vocal sounds, music, and environmental sounds, as well as auditory scene analysis. To gain a broader view of what acoustic features support this ability across domains, we curated a diverse set of natural sounds within and across different sound categories that are relevant to human listeners. We then obtained an exhaustive set of dissimilarity ratings for each pair of these sounds. Multiple analyses converged to reveal that acoustic qualities relating to aperiodicity, spectral variability (either overall, or in terms of change over time for other measures), spectral envelope (including spectral centroid), and temporal envelope (temporal centroid), could most reliably account for how participants made their responses. We also note that sound identification data were only moderately correlated with the dissimilarity ratings, suggesting interesting differences between these tasks and data that future work might reconcile. Finally, our results suggest an influence of musical training, which was related to identification accuracy and perhaps led participants to rely less on acoustic features when making dissimilarity ratings. Together these findings can help inform the development of machine intelligence applications to identify sounds in the environment, and assistive technologies to help an aging population where hearing loss is a major impediment to daily life. Our musical training results suggest that training may modulate how participants identify and distinguish sounds, and (following further study) could open the door to new training-based therapies or early interventions to counteract the effects of hearing loss on natural sound perception.

## Ethics Statement

This study was carried out in accordance with the recommendations of the University of Maryland Institutional Review Board with written informed consent from all subjects. All subjects gave written informed consent in accordance with the Declaration of Helsinki. The protocol was approved by the University of Maryland Institutional Review Board.

## Author Contributions

MO and LS designed the study and wrote the manuscript. MO collected and analyzed the data.

## Conflict of Interest Statement

The authors declare that the research was conducted in the absence of any commercial or financial relationships that could be construed as a potential conflict of interest.
