# Supplementary material for: Acoustic Correlates of Auditory Object and Event Perception: Speakers, Musical Timbres, and Environmental Sounds
Source: Front Psychol. 2019 Jul 17;10:1594. doi: 10.3389/fpsyg.2019.01594 (PMC6650748; doi:10.3389/fpsyg.2019.01594)
Supplement: Supplementary file 1 [file Data_Sheet_1.PDF]

As a complement to the correlation-based analyses reported in the manuscript, we also conducted an all-subsets-regression model comparison analysis. The model comparison analysis (also similar to RSA) used linear mixed-effect models of trial-level dissimilarity ratings. We drew inspiration from representational similarity analysis techniques (Kriegeskorte and Kievit, 2013) and compared a series of linear mixed-effect regression models where the outcome variable was the trial-level dissimilarity responses from each subject. The predictors in these models were pairwise acoustic feature differences (see Table 1) between the stimuli on each trial (see Sell et al., 2015; Siedeburg et al., 2016 or Wedin and Goude, 1972 for similar regression-based approaches). These pairwise differences were calculated for each feature by taking the absolute value of the difference of that feature's value between each stimulus pair. This analysis also examined multi-value representations of the stimuli such as for modulation power spectra and ERB cochleagram representations (medians and IQRs calculated for each ERB channel across time). Pairwise differences for these representations were obtained by calculating the Euclidean distance among the set of values in each representation between each stimulus pair (see 3.1.4, Table 1 and Figure 4). All of the acoustic predictors and outcome measures (dissimilarity ratings) were rank-transformed and then z-normalized in the models. Models also contained random intercepts for subjects and random intercepts for each of the unique 630 item pairs (since the pairwise acoustic feature differences are the same regardless of the order of the stimuli in the pair).

The aim of the linear mixed-effects model comparison analysis was to identify the set of features participants used to distinguish among pairs of items (not unlike identifying features related the dimensions in an MDS space). Thus, we performed an all-subsets regression model comparison analysis using in the "MuMIn" package (version 1.40.0, Bartoń, 2017) in R. This analysis fits a series of linear mixed-effect models containing all possible combinations of our acoustic predictors (pairwise differences between items for each acoustic feature) and ranks these models by their fit to the data. Because our aim here was essentially exploratory, we emphasized parsimony in our interpretation of these results in a number of ways. First, using simpler models (obtained via all-subsets regression) rather than simply using a full model that includes all predictors helps avoid over-fitting that might arise when including as many predictors as possible (Myung, 2000). Second, we used a BIC criterion to assess model fit (rankings), which emphasizes parsimony via strong penalties against the inclusion of additional model predictors (Murtaugh, 2009; Wagenmakers, 2007). The use of a BIC criterion also helps avoid type-I inflation errors associated with *p*-values, R-squared criteria, and step-wise procedures (Whittingham et al., 2006). Finally, to avoid potential biases associated with selecting a single top ranked model, when comparing against models that also might perform well (Burnham and Anderson, 2004), we report the results of our model-comparison procedure using a final model-averaging step. This aggregates the values of the predictors among the best performing models (within 3 BIC of the top ranked model).

Finally, our modeling approach allows for an intuitive examination of the potential influence of musical training on dissimilarity rating responses. After identifying the set of features that best fit the data through the model comparison and averaging analysis, we performed a second all-subsets regression and averaging analysis that also included the

interaction of the (rank-transformed and z-normalized) musical training subscale of the Gold-MSI questionnaire with each of the features included in the top performing (averaged) models. Again, to encourage parsimony and to guard against over fitting, the interaction was only examined if both the main effects (the feature and musical training predictors) were also included in the model.

All-subsets regression results

This model comparison analysis is summarized in Supplemental Table 1 and Supplemental Figures 1 and 2 (with the full models summarized in Supplemental Table 2). Model predictors (acoustic feature differences) were rank-transformed and z-normalized so the coefficients can be interpreted similar to a (rank-order) correlation strength between differences along a given acoustic feature dimension and the resulting dissimilarity rating (i.e., a larger acoustic difference for a feature is associated with a higher dissimilarity ratings).

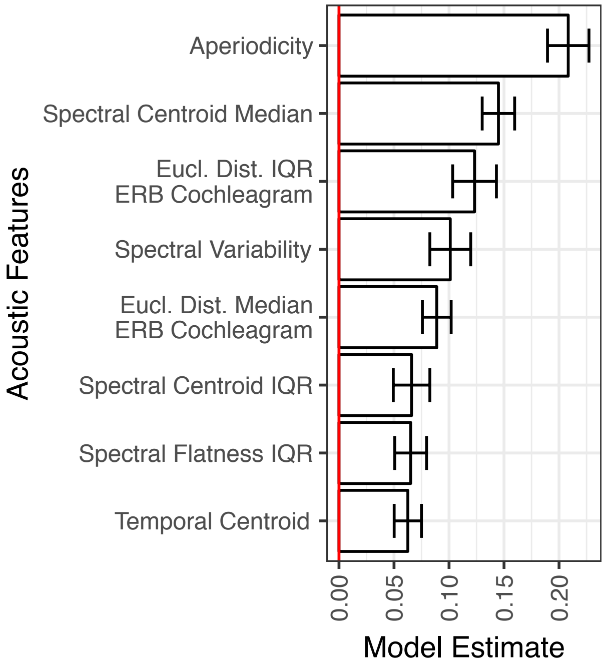

Supplemental Figure 1. Coefficients and standard errors of models of dissimilarity rating responses (averaged within 3 BIC of top-ranked model). Model estimates were z-normalized so the magnitudes of the coefficients are comparable. Positive model coefficients indicate that larger differences among stimulus pairs along that dimension are associated with higher dissimilarity ratings.

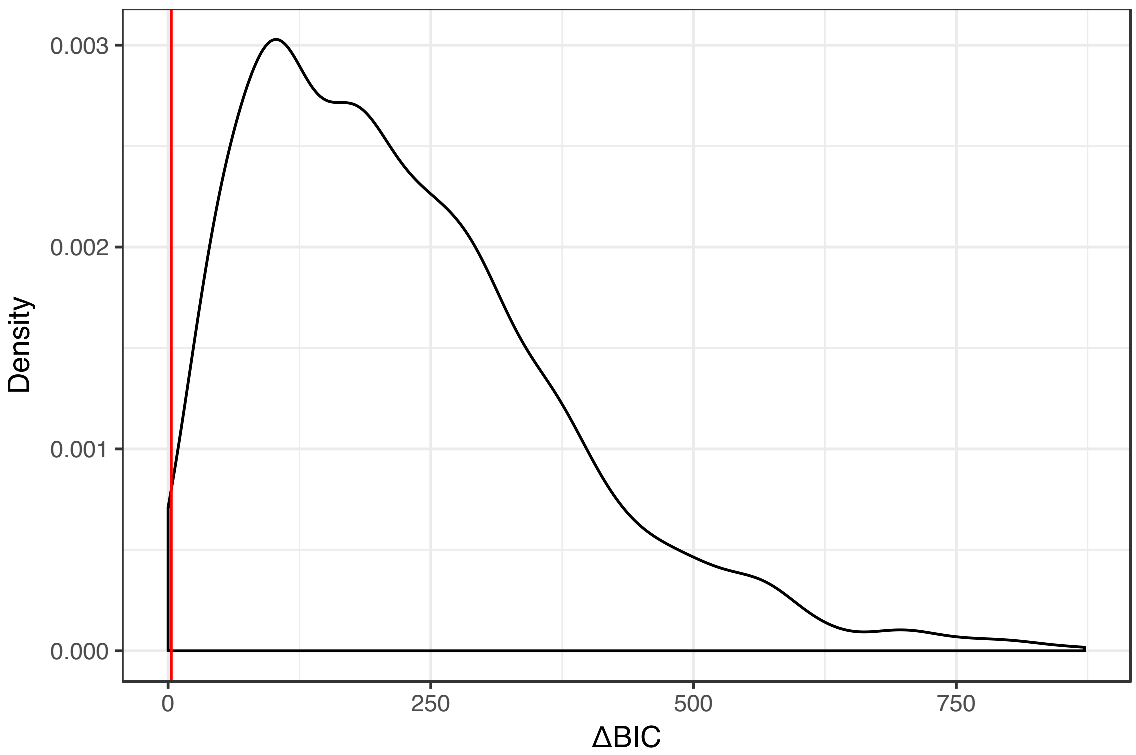

Supplemental Figure 2. Distribution of the BIC model fit scores for the models in the all-subset regression analysis. BIC scores are plotted relative to the top-ranked model (x-axis). The red vertical line indicates the 3-BIC cutoff used in the model-averaging step.

Supplemental Table 1. Models Comparison Analysis of Dissimilarity Ratings: Averaged Model Results Within 3 BIC

Parameters	Beta	SE	z	p	VIF	Importance
(Intercept)	0	0.05	0	1	NA	N/A
Aperiodicity	0.21	0.02	11.04	< 0.001	2.66	1
Eucl. Dist. IQR ERB Cochleagram	0.12	0.02	6.2	< 0.001	2.9	1
Eucl. Dist. Median ERB Cochleagram	0.09	0.01	6.79	< 0.001	1.37	1
IQR Spectral Centroid	0.07	0.02	3.97	< 0.001	1.93	0.41
IQR Spectral Flatness	0.07	0.01	4.5	< 0.001	1.55	0.79
Median Spectral Centroid	0.14	0.01	9.82	< 0.001	1.58	1
Spectral Variability	0.1	0.02	5.45	< 0.001	2.3	1
Temporal Centroid	0.06	0.01	5.04	< 0.001	1.2	1

Formula: (Dissimilarity Response) ~ (Aperiodicity) + … (Temporal Centroid) + (1| Subject) + (1|Item Pair)
Dissimilarity response and acoustic feature variables were rank-transformed and z-normalized in the model.
VIF calculated based on a non-averaged linear mixed-effect model containing all top predictors.
Importance indicates how frequently/highly a predictor appeared in the top ranked models.
R^2 for the fixed effects of a model containing these top-ranked features = 29.6%.

The top ranked models contained a similar set of features as the MDS solution reported in the main text: aperiodicity, spectral variability (along with the IQR of spectral centroid, spectral flatness and the ERB cochleagram), spectral envelope (both differences in ERB

cochleagram and spectral centroid), and a measure of the temporal envelope (temporal centroid). To understand how these results might be influenced by potential collinearity among the features, we calculated variance inflation factors (VIF; derived via: https://github.com/aufrank/R-hacks/blob/master/mer-utils.R) using a single linear mixed-effect model that contained the top-ranked features as predictors (Supplemental Table 1). These VIFs were quite low, especially compared to the typical employed cut-off for concerning levels of collinearity (VIF > 10; Cohen et al., 2003). The model that contained all the top-ranked features also accounted for 30% of the variance in participant responses (R^2 for the fixed effects in the model = 29.6%). Finally, relative importance values from the all-subsets regression were examined, which indicate how frequently a given predictor was included among the top ranked (averaged) models and where those models fell in the ranking (a feature that was in all of the top-ranked models would have an importance of 1). This suggests that the IQR of spectral centroid and of spectral flatness were not as high performing as the other features that were included.

Supplemental Table 2. Full Model Results of Dissimilarity Ratings for Experiment 2

**Parameters**	**Beta**	**SE**	**t**	**p**	**VIF**
(Intercept)	0	0.05	0	1	N/A
Aperiodicity	0.2	0.02	10.43	< 0.001	3.16
Eucl. Dist. IQR ERB Cochleagram	0.12	0.02	6.39	< 0.001	3
Eucl. Dist. Median ERB Cochleagram	0.09	0.01	6.8	< 0.001	1.43
Eucl. Dist. Modulation Power Spectrum	-0.01	0.01	-0.86	0.39	1.83
IQR Spectral Centroid	0.05	0.02	3.49	< 0.001	1.98
IQR Spectral Flatness	0.05	0.01	3.91	< 0.001	1.56
Log-Attack-Time	0	0.02	-0.14	0.89	1.98
Median Spectral Centroid	0.16	0.02	10.3	< 0.001	1.95
Median Spectral Flatness	-0.04	0.01	-2.98	< 0.001	1.21
Spectral Variability	0.09	0.02	5.47	< 0.001	2.36
Temporal Centroid	0.06	0.01	4.34	< 0.001	1.62

Formula: (Dissimilarity Response) ~ (Aperiodicity) + … (Temporal Centroid) + (1| Subject) + (1|Item Pair)
Dissimilarity response and acoustic feature variables were rank-transformed and z-normalized in the model.
VIF calculated based on a non-averaged linear mixed-effect model containing all predictors.
R^2 for the fixed effects of a model containing these top-ranked features = 29.7%.

Results of exploring the potential influence of musical training on models of participant dissimilarity responses

We recruited participants with a range of musical backgrounds. Given the influence of musical training on identification (Experiment 1), and previous results that suggest an influence of musical training on auditory object and event perception (Chartrand, and Belin, 2006; Tierney et al., 2015), we assessed how musical training influenced task performance and the use of different acoustic features. To do this, we took the top performing predictors from the all-subsets regression analysis and performed another all-subsets regression that also included the (rank-transformed and z-normalized) musical

training subscale of the Gold-MSI and its interaction with the top performing acoustic features. This allowed us to determine if including musical training and its interaction with the acoustic features provided an improvement to model fit.

The top-ranked models from this analysis were also averaged and are summarized in Supplemental Table 3. The same set of top ranked acoustic features was obtained. However, including musical training and its interaction with certain acoustic features improved the model fit despite the strong penalties imposed by the BIC criteria for the inclusion of additional features. Interestingly, a higher degree of musical training (which did not significantly influence responses on its own) tempered the influence of aperiodicity, and spectral envelope (both the ERB cochleagram representation and spectral centroid) via negative interactions. This suggests that more musically experienced participants relied less on these acoustic cues when making dissimilarity judgments.

Supplemental Table 3. Models Comparison Analysis of Dissimilarity Ratings: Influence of Musical Training on Averaged Model Results Within 3 BIC of Top

**Parameters**	**Beta**	**SE**	**z**	**p**	**VIF**	**Importance**
(Intercept)	0	0.05	0	1	NA	NA
Aperiodicity	0.21	0.02	11.04	< 0.001	2.66	1
Aperiodicity * Musical Training	-0.02	0	4.31	< 0.001	1.09	0.4
Eucl. Dist. IQR ERB Cochleagram	0.12	0.02	6.2	< 0.001	2.9	1
Eucl. Dist. Median ERB Cochleagram	0.09	0.01	6.79	< 0.001	1.37	1
IQR Spectral Centroid	0.07	0.02	3.97	< 0.001	1.93	0.41
IQR Spectral Flatness	0.07	0.01	4.5	< 0.001	1.55	0.79
Median Spectral Centroid	0.14	0.01	9.82	< 0.001	1.58	1
Median Spectral Centroid * Musical Training	-0.03	0	6.65	< 0.001	1.09	1
Musical Training	0	0.05	0.08	0.94	1	1
Spectral Variability	0.1	0.02	5.45	< 0.001	2.3	1
Temporal Centroid	0.06	0.01	5.04	< 0.001	1.2	1

Random effects structure was the same as Table 2
Dissimilarity response and acoustic feature variables were rank-transformed and z-normalized in the model.
VIF calculated based on a non-averaged linear mixed-effect model containing all top predictors.
Importance indicates how frequently/highly a predictor appeared in the top ranked models.
R^2 for the fixed effects of a model containing these top-ranked features = 29.7%.

References

Burnham, K. P., and Anderson, D. R. (2004). Multimodel inference understanding AIC and BIC in model selection. *Sociol. Methods Res.* 33, 261–304.

Chartrand, J.-P., and Belin, P. (2006). Superior voice timbre processing in musicians. *Neurosci. Lett.* 405, 164–167.

Cohen, H., Cohen, O., West, S. G., and Aiken, L. S. (2003). *Applied Multiple Regression/Correlation Analysis for the Behavioral Sciences, 3rd Edn.* LEA.

Kriegeskorte, N., and Kievit, R. A. (2013). Representational geometry: integrating cognition, computation, and the brain. *Trends Cogn. Sci.* 17, 401–412.

Murtaugh, P. A. (2009). Performance of several variable-selection methods applied to real ecological data. *Ecol. Lett.* 12, 1–8.

Myung, I. J. (2000). The importance of complexity in model selection. *J. Math. Psychol.* 44, 190–204.

Sell, G., Suied, C., Elhilali, M., and Shamma, S. (2015). Perceptual susceptibility to acoustic manipulations in speaker discrimination. *J. Acoust. Soc. Am.* 137, 911–922.

Siedenburg, K., Jones-Mollerup, K., and McAdams, S. (2016). Acoustic and categorical dissimilarity of musical timbre: evidence from asymmetries between acoustic and chimeric sounds. *Front. Psychol.* 6:1977.

Tierney, A. T., Krizman, J., and Kraus, N. (2015). Music training alters the course of adolescent auditory development. *Proc. Natl. Acad. Sci. U.S.A.* 112, 10062–10067.

Wagenmakers, E. J. (2007). A practical solution to the pervasive problems of p values. *Psychon. Bull. Rev.* 14, 779–804.

Wedin, L., and Goude, G. (1972). Dimension analysis of the perception of instrumental timbre. *Scand. J. Psychol.* 13, 228–240.

Whittingham, M. J., Stephens, P. A., Bradbury, R. B., and Freckleton, R. P. (2006). Why do we still use stepwise modelling in ecology and behaviour? *J. Anim. Ecol.* 75, 1182–1189.
